# The impact of public insurance on RRSO for HBOC in Japan: a nationwide data study

**DOI:** 10.1038/s10038-025-01326-0

**Published:** 2025-05-21

**Authors:** Megumi Matsumoto, Hiroki Den, Shoko Miura, Ayumi Harada, Hiroyuki Nomura, Masami Arai, Hiraku Kumamaru, Seigo Nakamura, Masayuki Sekine, Kiyonori Miura

**Affiliations:** 1 Department of Breast Endocrine Surgery, Japanese Red Cross Nagasaki Genbaku Hospital, Nagasaki, Japan; 2 Department of Hygiene, Public Health, and Preventative Medicine, Showa University School of Medicine, Tokyo, Japan; 3 Department of Obstetrics and Gynecology, Nagasaki University Graduate School of Biomedical Sciences, Nagasaki, Japan; 4 Department of Obstetrics and Gynecology, Nagasaki University Hospital, Nagasaki, Japan; 5 Department of Obstetrics and Gynecology, Tokai University School of Medicine, Isehara, Japan; 6 Department of Clinical Genetics, Juntendo University, Graduate School of Medicine, Tokyo, Japan; 7 Department of Healthcare Quality Assessment, The University of Tokyo Graduate School of Medicine, Tokyo, Japan; 8 Division of Breast Surgical Oncology, Department of Surgery, Showa University School of Medicine Institute for Clinical Genetics and Genomics Showa University, Tokyo, Japan; 9 Department of Obstetrics and Gynecology, Graduate School of Medical Science University of the Ryukyus, Okinawa, Japan

**Keywords:** Cancer genetics, Gynaecological cancer

## Abstract

In Japan, risk management based on genetic disposition, such as risk-reducing surgery for hereditary breast and ovarian cancer (HBOC), is covered by public insurance only for some cancer patients. However, non-cancer clients are forced to pay a high co-payment. In this study, we examined the impact of risk-reducing salpingo-oophorectomy (RRSO), before and after the April 2020 public insurance coverage, using nationwide data from the Japanese Organization of Hereditary Breast and Ovarian Cancer. The period from March 2006 to March 2020 was defined as before insurance coverage (Pre), and the period from April 2020 to August 2021 was defined as after insurance coverage (Post). In addition, the period from April 2018 to March 2020 was designated as special (short-Pre) to coincide with the post-insurance coverage period. Of the 383 breast cancer patients who underwent genetic testing at Short-Pre, 42 (11.0%) underwent RRSO during that period. Of the 623 breast cancer patients who underwent genetic testing at Post, 142 (22.8%) underwent RRSO during that period. Significantly, this comparison shows an increase in RRSO rates in Post. Separating *BRCA1* and *BRCA2* also significantly increased RRSO in Post. This nationwide survey suggests that if RRSO is covered by insurance in Japan, the implementation rate will increase. As the number of cases increases in the future, the impact of insurance coverage will become clearer. If the insurance coverage for RRSO in Japan is determined to be useful, this information can be used to expand coverage for those who have not yet developed the disease.

## Introduction

Hereditary breast and ovarian cancer (HBOC) is among the most common hereditary tumors and is known to be inherited in an autosomal dominant form, involving the *BRCA1* and *BRCA2* genes [1, 2]. The percentage of *BRCA* gene pathological variant carriers in the general Japanese population is reportedly 0.21% [3]. The efficacy of Poly (ADP-ribose) polymerase (PARP) inhibitors in the treatment of patients with cancer with known *BRCA* pathological variants has resulted in the use of the *BRCA* gene as a companion diagnosis [4–7]. Following the Food and Drug Administration’s approval of PARP inhibitors (Olaparib) for treating ovarian cancer in 2014, the drug was initially covered by public insurance in Japan in April 2018 for recurrent ovarian cancer, followed by the extension of coverage to inoperable or recurrent breast cancer, metastatic prostate cancer, and pancreatic cancer not curatively resectable [8–10]. In addition, the opportunity for HBOC to be diagnosed is increasing because cancer gene panel testing has also been covered by public insurance since 2019 [11].

Risk-reducing salpingo-oophorectomy (RRSO) for *BRCA* pathological variant carriers has been recommended by various guidelines [9, 12], because it prevents the development of ovarian and fallopian tube cancers, prolongs overall survival [13–19], and reduces cancer mortality through risk-based health management by identifying genetic disposition. In April 2020, in addition to genetic counseling, surveillance, and risk-reducing surgery, *BRCA* genetic testing (GT) for diagnosing HBOC was also covered by public insurance for some patients with breast and all patients with ovarian cancers in Japan. Although the results of this GT for cancer diagnosis and treatment is expected to lead to future diagnoses of HBOC, as well as an increase in blood-relative diagnoses, public insurance coverage is currently limited to patients with breast and ovarian cancer. Those without cancer are currently forced to pay their own expenses [20]. This study examined the current status of RRSOs in Japan and the impact of public insurance coverage by determining behavioral changes before and after public insurance coverage in April 2020.

## Materials and methods

### Study registration

The data used in this study were obtained from data registered with the Japanese Organization of HBOC (JOHBOC) [21]. JOHBOC has a nationwide database based on families that have undergone *BRCA* GT. In the JOHBOC database, medical institutions register probands that received *BRCA1* and/or *BRCA2* GT and their relatives. Any purposes for GT are acceptable, including for clinical practice and translational research. Almost all GT, including sequence and large rearrangement analysis, is performed at Myriad Genetic Laboratories or FALCO Biosystems. The detected variants are interpreted according to the criteria followed at Myriad Genetic Laboratories. Previous studies have outlined details regarding the registration procedures [22, 23]. They have also identified relevant factors related to HBOC and given recommendations to women with HBOC in the guidelines. This study used the following data: number of germline *BRCA1/2* mutants; number of RRSO; duration from GT to RRSO (comparing before and after insurance coverage); number and age of patients with first-time breast cancer who underwent RRSO; breast cancer onset and timing of RRSO; duration to RRSO divided by breast cancer onset age; timing of RRSO and risk-reducing mastectomy (RRM) for breast cancer patients; background of clients who received GT (comparing before and after insurance coverage); and RRSO rate (comparing before and after insurance coverage).

Informed consent was obtained from all participants at the time of enrollment in JOHBOC. In cases where informed consent could not be obtained in person, such as those wherein treatment had been completed or the patient had died, the candidate or the candidate’s family could opt out of the JOHBOC and each participating institution’s website. All patients received genetic counseling and GT of their own free will. Informed consent for this study disclosed that the study was being conducted with opt out as designated by a paper on the JOHBOC website.

To analyze how each survey item was affected by insurance coverage as of April 2020, when HBOC treatment was included under insurance coverage in Japan, the period from March 2006 to March 2020 was defined as pre-insurance coverage (Pre), and that from April 2020 to August 2021 was defined as post-insurance coverage (Post). As this study compared the impact of insurance coverage on RRSOs, the difference between Pre (14 years) and Post (1 year and 5 months) were considered significant. To establish a period equivalent to Post when assessing the impact of insurance coverage, the short period before insurance coverage (short-Pre) was defined as the 2 years immediately preceding April 2018 to March 2020. For each period, the applicable cases were those that underwent GT and RRSO within that period.

### Statistical analyses

A two-sample t-test was conducted to compare the means of the two groups. Regarding the categorical variables, a chi-squared test was conducted as a test of independence. Additionally, a Wilcoxon rank sum test was performed to compare the two groups’ medians. Further, a Kruskal–Wallis rank sum test was used to compare the medians of the three groups. A two-sample test for equality of proportions was conducted to compare the proportions of two groups. Considering the small sample size and low frequency of events, statistical analysis was performed using Fisher’s exact test to compare the proportions between the two groups. The significance level was set at *α* = 0.05. All statistical analyses were performed using R programming version 4.1.2 (R Development Core Team, Vienna, Austria).

## Results

**Fig. 1:**
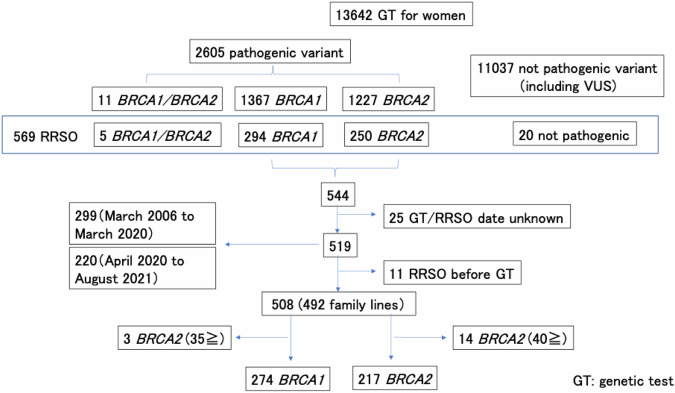
Flowchart of selection of eligible studies

A total of 13,642 women who had undergone BRCA GT between March 2006 and August 2021 were included in JOHBOC. Of the 2605 pathogenic variants, this study included 544 cases of either BRCA1 or BRCA2 variants that underwent RRSO. Among them, 299 RRSOs were performed from March 2006 to March 2020 and 220 from April 2020 to August 2021, except for 25 cases for which the date of examination or the date of RRSO was unknown. Ultimately, 508 cases were included, excluding 11 cases that received GT after the RRSO date (Fig. 1). First, we analyzed the “background factors influencing the decision to conduct an RRSO” for the entire study period, and then compared the “impact of insurance coverage on RRSO” before and after public insurance coverage was applied to RRSO.

### Duration from GT to RRSO

**Fig. 2:**
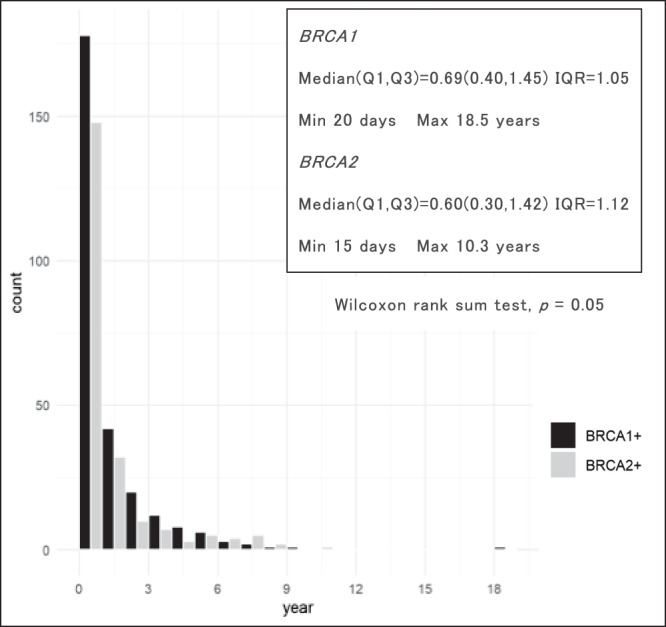
Duration from GT to RRSO for *BRCA1* and *BRCA2,* respectively

For the period March 2006 to August 2021, the median time from GT to RRSO for all cases was 0.66 years (15 days to 18.5 years) (Supplementary Fig. S1). When *BRCA1* and *BRCA2* were examined separately, *BRCA2* tended to have a shorter time from GT to RRSO (Wilcoxon rank sum test, *p* = 0.05) (Fig. 2).

### Breast cancer onset and timing of RRSO

Of the 508 patients who had undergone RRSO after GT, 476 (93.7%) had breast cancer. Regarding the timing of RRSO, RRSO was performed in intervals after breast cancer surgery (more than 2 weeks) in 414 cases (87.0%), and at approximately the same time as breast cancer surgery (within 2 weeks) in 57 cases (12.0%). Additionally, four patients (0.8%) developed breast cancer after RRSO and were operated on, and one patient (0.2%) had not provided details on the timing.

### Duration to RRSO divided by breast cancer onset age

**Fig. 3:**
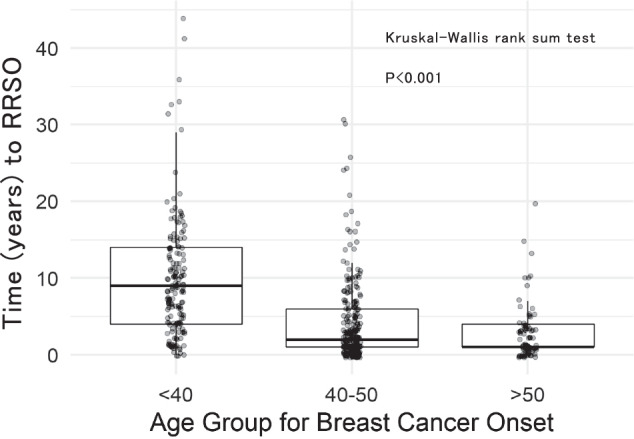
Duration to RRSO divided by breast cancer onset age

This survey was conducted between March 2006 and August 2021. The median time to RRSO was 8 years for individuals <40 years, 3 years for those 40–50 years, and 2 years for those ≥51 years, indicating that the time to RRSO was longer for younger age groups (<40 years) at breast cancer’s first onset (Kruskal–Wallis rank sum test, *p* < 0.001) (Fig. 3).

### Timing of RRSO and RRM for breast cancer patients

The timing of surgery in the 127 cases in which both RRSO and RRM were performed was evaluated. Of these, 69 cases (54.3%) had both procedures performed within a 1-month interval, 31 cases (24.4%) had RRSO followed by RRM, and 27 cases (21.3%) had RRSO followed by RRM. The majority of patients underwent simultaneous RRM and RRSO. Details of the timing of surgery are shown in Supplementary Table S1, pre and post, *BRCA1* and *BRCA2*, respectively.

### Impact of insurance coverage on GT

This study compared a 2-year period from April 2018 to March 2020 (short-Pre) with a 1-year and 5-month period from April 2020 to August 2021 (Post). Table 1 presents the participants’ backgrounds. No significant differences in background were identified in short-Pre and Post, including the ratio of *BRCA1* and *BRCA2*, average age at GT, age groups at GT, and history of cancer. However, significant differences were identified in the purpose for GT (multiple choice): chemotherapy options (*p* < 0.01) and research (*p* < 0.01) were often the purpose for GT in short-Pre, whereas individuals’ healthcare (*p* < 0.01) and surgical decisions (*p* < 0.01) were often the purpose for GT in Post. No difference was identified in family purpose between short-Pre and Post (*P* = 0.21).

**Table 1:** Background of clients who received GT

	short-Pre	Post	*P*
*N*	641	913	
BRCA1+/BRCA2+	323(50.4%)/318(49.6%)	416(45.6%)/497(54.4%)	0.07
Average age at GT (SD)	49.4 (13.0)	50.0 (12.6)	0.39
Age group			
<40	138	162	0.09
40–45	92	165	
45–50	135	179	
51<	276	407	
At the time of GT			
History of breast cancer	463 (72.2%)	691 (75.7%)	0.14
History of ovarian cancer	154 (24.0%)	197 (21.6%)	0.28
History of pancreatic cancer	1 (0.16%)	2 (0.22%)	–
Purpose for GT (multiple choice)			
Health care of the individual	322 (50.2%)	635 (69.6%)	*p* < 0.01
For the family	176 (27.5%)	279 (30.6%)	*p* = 0.21
Surgical decision	52 (8.1%)	158 (17.3%)	*p* < 0.01
Chemotherapy options	239 (37.3%)	214 (23.4%)	*p* < 0.01
Research	48 (7.5%)	6 (0.7%)	*p* < 0.01

### RRSO rates before and after insurance coverage

Of the 641 patients who underwent GT in short-Pre, 383 breast cancer patients were included, excluding ovarian cancer patients and those who underwent RRSO during Post. Similarly, of the 913 patients who underwent GT in Post, 623 breast cancer patients were included, excluding ovarian cancer patients and those who underwent RRSO at or below the recommended age (35 years for *BRCA1* and 40 years for *BRCA2*). A significant difference was observed between the two groups (two-sample test for equality of proportions, *p* < 0.01). Furthermore, RRSO rates at Post increased significantly when *BRCA1* and *BRCA2* were evaluated, respectively (two-sample test for equality of proportions, *p* < 0.01 for *BRCA1* and *BRCA2)*. In addition, the cases of no breast cancer and no ovarian cancer were also examined. Contrary to breast cancer patients, the RRSO rate was significantly higher (*p* = 0.03) for short-Pre among these cases (Table 2).

**Table 2:** “Breast cancer patient” is evaluated by 2-sample test for equality of proportions, but ‘No Breast cancer and no Ovarian cancer’ is evaluated by Fisher 's exact test

	short-Pre		Post		*P*
	*N*	RRSO	*N*	RRSO	
**Breast cancer patient (no ovarian cancer)**					
Total	383	42 (11.0%)	623	142 (22.8%)	<0.01
*BRCA1* (35≦)	170	23 (13.5%)	243	65 (26.8%)	<0.01
*BRCA2* (40≦)	213	19 (8.9%)	380	77 (20.3%)	<0.01
**No breast cancer and no ovarian cancer**					
Total	63	9(14.29%)	87	3 (3.45%)	0.03
*BRCA1* (35≦)	38	8(21.1%)	48	3 (6.25%)	0.05
*BRCA2* (40≦)	25	1(4%)	39	0 (0%)	0.39

2-sample test for equality of proportions

Fisher’s exact test

### Duration from GT to RRSO before and after insurance coverage

There were no significant differences in median time from GT to RRSO t before and after insurance coverage (Short-Pre: Median (Q1,Q3) = 177.5 (108.8,251.0) IQR = 142.2, Post: Median (Q1,Q3) = 143 (93.3,215.3) IQR = 122. Wilcoxon rank sum test, *p* = 0.11).

## Discussion

HBOC practice aims to reduce mortality from related cancers by identifying patients’ genetic makeup and providing risk-based healthcare [24, 25]. This study suggests that the prognosis can be improved by performing GT on appropriate individuals and promoting treatment, prevention, and surveillance suited to their constitutions. However, the environment in Japan is not yet conducive to providing RRSO to all applicants. In April 2020, RRSO and RRM was added to coverage for some patients with breast cancer and for all patients with ovarian cancer. This was a major paradigm shift; however, many eligible patients who wish to undergo risk-reducing surgery, including those who have not yet developed the disease, must pay their own high expenses [21]. Although psychosocial factors such as cultural norms, social values, and the attitudes of healthcare providers are intricately involved in RRSO decision-making [26], this study evaluated the status of RRSO and examined the impact of insurance coverage by reviewing data before and after insurance coverage. One of this study’s strengths was that the analysis was based on a Japanese nationwide database in JOHBOC.

This study first examined the entire period from GT to RRSO (March 2006 to August 2021), during which time HBOC practice in Japan changed significantly. For most of this period, RRSO was expensive, with a median time from GT to RRSO of 241 days (0.66 years) (Supplementary Fig. S1). When *BRCA1* and *BRCA2* were examined separately, *BRCA2* showed a trend toward a shorter time from GT to RRSO (Wilcoxon rank sum test, *p* = 0.05) (Fig. 2). By contrast, to examine the impact of insurance, short-Pre and Post were compared separately, but there was no significant difference in the time from GT to RRSO.

In breast cancer patients, the RRSO implementation rates before and after insurance coverage were examined separately for short-Pre and Post, the short-Pre RRSO implementation rate was 11.0% and the Post RRSO implementation rate was 22.8%, revealing a considerable difference. The same was true when RRSO rates were examined separately for *BRCA1* and *BRCA2*, with significantly increased RRSO rates in Post. We simultaneously added data for those with no breast cancer and no ovarian cancer and found that the RRSO rate was higher for short-Pre with a significant difference (Table 2). As a result, breast cancer patients whom RRSO could be performed by insurance had significantly higher RRSO rates in Post. Additionally, those with no breast cancer and no ovarian cancer whom RRSO was not covered by insurance, had significantly higher RRSO rates in short-Pre. It is unclear why the RRSO rate was higher for short-Pre in those with no breast cancer and no ovarian cancer, but the rate did not increase after insurance coverage. Thus, if breast cancer patients are considered the insured group and those with no breast cancer and no ovarian cancer are considered the co-payments group, the decrease in co-payments could have led to an increase in the rate of RRSO implementation.

This study also examined the background of clients tested during short-Pre and Post (Table 1). Significant differences between the short-Pre and Post periods were found regarding the purpose of GT. The common objectives for being tested during short-Pre were chemotherapy options and research, while the common objectives for undergoing GT during Post were healthcare of the individual and surgical decision. The reason that chemotherapy options were often selected as the purpose of GT during short-Pre was presumably because *BRCA* had been used as a companion diagnosis for breast and ovarian cancer in Japan since 2018. Additionally, this study suggested that the reason for undergoing GT during short-Pre was often research, to avoid the high private expenses. However, the significant increase in GT during Post due to the healthcare of the individual and surgical decision may be due to the inclusion of insurance coverage in 2020. If insurance coverage of *BRCA* is expanded to cancer-naïve clients in the future, more individuals are expected to request GT. One bias to consider, as Table 1 shows, is the occurrence of chemotherapy after breast cancer surgery in some cases. It is inferred that there are cases where the Post has received GT but not yet received RRSO because the Post period was of a short duration.

Since patients who request RRSO are often assumed to be breast cancer patients, this study examined the association between breast cancer patients and RRSO. In this study, 93.7% of the patients who underwent RRSO were breast cancer patients; 87.0% of breast cancer patients underwent RRSO at a different time from breast cancer surgery. RRSO is covered by insurance now, and the number of cases in which RRSO is performed simultaneously with breast cancer surgery may increase in the future [27].

When the time from breast cancer onset to RRSO was compared separately by age of breast cancer onset, the time for patients younger than 40 years tended to be significantly longer (Fig. 3). Additionally, unique circumstances of young adults (<40), such as pregnancy, childbirth, and childcare, may also result in a longer interval before RRSO. However, in the group of patients <40 years of age, who are presumed to be economically disadvantaged, the period may be shortened in the future owing to the introduction of insurance treatment. The European Society for Medical Oncology (ESMO) guidelines generally recommend that RRSO should be performed between 35 and 40 years of age, while the National Comprehensive Cancer Network (NCCN) guidelines recommend it between 35 and 40 years of age for *BRCA1* and between 40 and 45 years of age for *BRCA2*. In the group of patients <40 years of age, the time to RRSO is expected to be longer if the disease occurs at a younger age. As RRSO and RRM were expensive procedures before insurance coverage, the timing of each risk-reducing procedure was evaluated. In this study, both RRSO and RRM were performed in 127 cases, with over half of these cases opting for simultaneous RRSO and RRM. Performing both procedures simultaneously when they were not covered by insurance would have required the patient to pay a high out-of-pocket cost at once. If insurance coverage becomes available in the future, it is expected that more and more patients will request both surgeries simultaneously (Supplementary Table S1).

In conclusion, this nationwide study shows that insurance coverage of RRSOs in Japan has increased the RRSO implementation rate. Due to the short period of time since the RRSO was covered by insurance only for patients with some indications in this study, data are insufficient to show future trends. However, as the number of cases increases in the future, the impact of insurance coverage will become clearer. If the usefulness of insurance coverage for RRSOs is found in Japan, we hope that this information will be used to expand insurance coverage for those who have not yet developed the disease.

## Supplementary information

Supplementary Information

ICMJE Disclosure Form - AH

ICMJE Disclosure Form - HD

ICMJE Disclosure Form - HK

ICMJE Disclosure Form - HN

ICMJE Disclosure Form - KM

ICMJE Disclosure Form - MA

ICMJE Disclosure Form - MM

ICMJE Disclosure Form - MS

ICMJE Disclosure Form - SM

ICMJE Disclosure Form - SN

## Data Availability

The datasets generated and analyzed in this study are not publicly available because they deal with genomic information but are available upon reasonable request to The Japanese HBOC Consortium (http://hboc.jp/) from the author.

## References

[1] Petrucelli N, Daly MB, Feldman GL. Hereditary breast and ovarian cancer due to mutations in *BRCA1* and *BRCA2*. Genet Med. 2010;12:245–59. 10.1097/GIM.0b013e3181d38f2f 20216074 10.1097/GIM.0b013e3181d38f2f

[2] Petrucelli N, Daly MB, Pal T. BRCA1- and BRCA2-Associated Hereditary Breast and Ovarian Cancer. 1998 Sep 4 [Updated 2023 Sep 21]. In: Adam MP, Feldman J, Mirzaa GM, et al., editors. GeneReviews® [Internet]. Seattle (WA): University of Washington, Seattle; 1993–2024. https://www.ncbi.nlm.nih.gov/books/NBK1247/20301425

[3] Momozawa Y, Iwasaki Y, Parsons MT, Kamatani Y, Takahashi A, Tamura C, et al. Germline pathogenic variants of 11 breast cancer genes in 7,051 Japanese patients and 11,241 controls. Nat Commun. 2018;9:4083 10.1038/s41467-018-06581-8 30287823 10.1038/s41467-018-06581-8 PMC6172276

[4] Robson M, Im SA, Senkus E, Xu B, Domchek SM, Masuda N, et al. Olaparib for metastatic breast cancer in patients with a germline BRCA mutation. N Engl J Med. 2017;377:523–33. 10.1056/NEJMoa1706450 28578601 10.1056/NEJMoa1706450

[5] Tutt ANJ, Garber JE, Kaufman B, Viale G, Fumagalli D, Rastogi P, et al. Adjuvant Olaparib for patients with BRCA1- or BRCA2-mutated breast cancer. N Engl J Med. 2021;384:2394–405. 10.1056/NEJMoa2105215 34081848 10.1056/NEJMoa2105215 PMC9126186

[6] Mirza MR, Monk BJ, Herrstedt J, Oza AM, Mahner S, Redondo A, et al. Niraparib maintenance therapy in platinum-sensitive, recurrent ovarian cancer. N Engl J Med. 2016;375:2154–64. 10.1056/NEJMoa1611310 27717299 10.1056/NEJMoa1611310

[7] Gonzalez-Martin A, Pothuri B, Vergote I, Christensen RD, Graybill W, Mirza MR, et al. Niraparib in patients with newly diagnosed advanced ovarian cancer. N Engl J Med. 2019;381:2391–402. 10.1056/NEJMoa1910962 31562799 10.1056/NEJMoa1910962

[8] Giri VN, Knudsen KE, Kelly WK, Cheng HH, Cooney KA, Cookson MS, et al. Implementation of germline testing for prostate cancer: Philadelphia Prostate Cancer Consensus Conference 2019. J Clin Oncol. 2020;38:2798–811. 10.1200/JCO.20.00046 32516092 10.1200/JCO.20.00046 PMC7430215

[9] National Comprehensive Cancer Network. NCCN Clinical Practice Guidelines in Oncology. Genetic/Familial High-Risk Assessment: Breast, Ovarian, and Pancreatic. 2022. https://www.nccn.org/professionals/physician_gls/pdf/genetics_bop.pdf. 10.6004/jnccn.2021.0001 33406487

[10] Golan T, Hammel P, Reni M, Van Cutsem E, Macarulla T, Hall MJ, et al. Maintenance Olaparib for germline BRCA-mutated metastatic pancreatic cancer. N Engl J Med. 2019;381:317–27. 10.1056/NEJMoa1903387 31157963 10.1056/NEJMoa1903387 PMC6810605

[11] Ueki A, Yoshida R, Kosaka T, Matsubayashi H. Clinical risk management of breast, ovarian, pancreatic, and prostatic cancers for BRCA1/2 variant carriers in Japan. J Hum Genet. 2023;68:517–26. 10.1038/s10038-023-01153-1 37088789 10.1038/s10038-023-01153-1

[12] Sessa C, Balmaña J, Bober SL, Cardoso MJ, Colombo N, Curigliano G, et al. Risk reduction and screening of cancer in hereditary breast-ovarian cancer syndromes: ESMO Clinical Practice Guideline. Ann Oncol. 2023;34:33–47. 10.1016/j.annonc.2022.10.004 36307055 10.1016/j.annonc.2022.10.004

[13] Finch A, Beiner M, Lubinski J, Lynch HT, Moller P, Rosen B, et al. Salpingo-oophorectomy and the risk of ovarian, fallopian tube, and peritoneal cancers in women with a BRCA1 or BRCA2 Mutation. JAMA. 2006;296:185–92. 10.1001/jama.296.2.185 16835424 10.1001/jama.296.2.185

[14] Finch AP, Lubinski J, Møller P, Singer CF, Karlan B, Senter L, et al. Impact of oophorectomy on cancer incidence and mortality in women with a BRCA1 or BRCA2 mutation. J Clin Oncol. 2014;32:1547–53. 10.1200/JCO.2013.53.2820 24567435 10.1200/JCO.2013.53.2820 PMC4026578

[15] Finkelman BS, Rubinstein WS, Friedman S, Friebel TM, Dubitsky S, Schonberger NS, et al. Breast and ovarian cancer risk and risk reduction in Jewish BRCA1/2 mutation carriers. J Clin Oncol. 2012;30:1321–8. 10.1200/JCO.2011.37.8133 22430266 10.1200/JCO.2011.37.8133 PMC3341145

[16] Kauff ND, Domchek SM, Friebel TM, Robson ME, Lee J, Garber JE, et al. Risk-reducing salpingo-oophorectomy for the prevention of BRCA1- and BRCA2-associated breast and gynecologic cancer: a multicenter, prospective study. J Clin Oncol. 2008;26:1331–7. 10.1200/JCO.2007.13.9626 18268356 10.1200/JCO.2007.13.9626 PMC3306809

[17] Kauff ND, Satagopan JM, Robson ME, Scheuer L, Hensley M, Hudis CA, et al. Risk-reducing salpingo-oophorectomy in women with a BRCA1 or BRCA2 mutation. N Engl J Med. 2002;346:1609–15. 10.1056/NEJMoa020119 12023992 10.1056/NEJMoa020119

[18] Domchek SM, Friebel TM, Singer CF, Evans DG, Lynch HT, Isaacs C, et al. Association of risk-reducing surgery in BRCA1 or BRCA2 mutation carriers with cancer risk and mortality. JAMA. 2010;304:967–75. 10.1001/jama.2010.1237 20810374 10.1001/jama.2010.1237 PMC2948529

[19] Rebbeck TR, Lynch HT, Neuhausen SL, Narod SA, van’t Veer L, Garber JE, et al. Prophylactic oophorectomy in carriers of BRCA1 or BRCA2 mutations. N Engl J Med. 2002;346:1616–22. 10.1056/NEJMoa012158 12023993 10.1056/NEJMoa012158

[20] Hirasawa A, Masuda K, Akahane T, Tsuruta T, Banno K, Makita K, et al. Experience of risk-reducing salpingo-oophorectomy for a BRCA1 mutation carrier and establishment of a system performing a preventive surgery for hereditary breast and ovarian cancer syndrome in Japan: our challenges for the future. Jpn J Clin Oncol. 2013;43:515–9. 10.1093/jjco/hyt036 23487443 10.1093/jjco/hyt036

[21] The Japanese HBOC Consortium. http://hboc.jp/. Accessed 23 March 2024.

[22] Nomura H, Sekine M, Yokoyama S, Arai M, Enomoto T, Takeshima N, et al. Clinical background and outcomes of risk-reducing salpingo-oophorectomy for hereditary breast and ovarian cancers in Japan. Int J Clin Oncol. 2019;24:1105–10. 10.1007/s10147-019-01456-4 31055694 10.1007/s10147-019-01456-4

[23] Arai M, Yokoyama S, Watanabe C, Yoshida R, Kita M, Okawa M, et al. Genetic and clinical characteristics in Japanese hereditary breast and ovarian cancer: first report after establishment of HBOC registration system in Japan. J Hum Genet. 2018;63:447–57. 10.1038/s10038-017-0355-1 29176636 10.1038/s10038-017-0355-1 PMC8716335

[24] Kotsopoulos J, Gronwald J, Huzarski T, Møller P, Pal T, McCuaig JM, et al. Bilateral oophorectomy and all-cause mortality in women with BRCA1 and BRCA2 sequence variations. JAMA Oncol. 2024. 10.1001/jamaoncol.2023.6937 10.1001/jamaoncol.2023.6937 PMC10905374 38421677

[25] Lubinski J, Kotsopoulos J, Moller P, Pal T, Eisen A, Peck L, et al. MRI surveillance and breast cancer mortality in women with BRCA1 and BRCA2 sequence variations. JAMA Oncol. 2024. 10.1001/jamaoncol.2023.6944 10.1001/jamaoncol.2023.6944 PMC10905376 38421676

[26] Park SY, Kim Y, Kim S. Factors associated with the decision to undergo risk-reducing salpingo-oophorectomy among women at high risk for hereditary breast and ovarian cancer: a systematic review. Korean J Women Health Nurs. 2020;26:285–99. 10.4069/kjwhn.2020.11.19 36312308 10.4069/kjwhn.2020.11.19 PMC9328615

[27] Nomura H, Abe A, Fusegi A, Yoshimitsu T, Misaka S, Murakami A, et al. Impact of the coverage of risk-reducing salpingo-oophorectomy by the national insurance system for women with BRCA pathogenic variants in Japan. Sci Rep. 2023;13:1018 10.1038/s41598-023-28304-w 36658289 10.1038/s41598-023-28304-w PMC9852267

