# Supplementary material for: The impact of public insurance on RRSO for HBOC in Japan: a nationwide data study
Source: J Hum Genet. 2025 May 21;70(8):421–6. doi: 10.1038/s10038-025-01326-0 (PMC12289518; doi:10.1038/s10038-025-01326-0)
Supplement: Supplementary file 1 — Supplementary Information [file 10038_2025_1326_MOESM1_ESM.docx]

**Supplementary Information for the article:**

**The impact of public insurance on RRSO for HBOC in Japan: A nationwide data study**

Megumi Matsumoto^1^, Hiroki Den^2^, Shoko Miura^3^, Ayumi Harada^4^, Hiroyuki Nomura^5^, Masami Arai^6^, Hiraku Kumamaru^7^, Seigo Nakamura^8^, Masayuki Sekine^9*^, Kiyonori Miura^3*^

^1^Department of Breast Endocrine Surgery, Japanese Red Cross Nagasaki Genbaku Hospital

^2^ Department of Hygiene, Public Health, and Preventative Medicine, Showa University School of Medicine

^3^ Department of Obstetrics and Gynecology, Nagasaki University Graduate School of Biomedical Sciences

^4^ Department of Obstetrics and Gynecology, Nagasaki University Hospital

^5^Department of Obstetrics and Gynecology, Tokai University School of Medicine, Isehara, 259-1193, Japan

^6^ Department of Clinical Genetics, Juntendo University, Graduate School of Medicine, Tokyo, Japan

^7^ Department of Healthcare Quality Assessment, The University of Tokyo Graduate School of Medicine

^8^Division of Breast Surgical Oncology, Department of Surgery, Showa University School of Medicine Institute for Clinical Genetics and Genomics Showa University, Tokyo, Japan

^9^Department of Obstetrics and Gynecology, Graduate School of Medical Science University of the Ryukyus

***Correspondence:**

Masayuki Sekine; Department of Obstetrics and Gynecology, Graduate School of Medical Science University of the Ryukyus, 207 Uehara, Nishihara, Okinawa 903-0215 Japan; Email: msekine@med.u-ryukyu.ac.jp; Phone: +81-98-895-1177; Fax: +81-98-895-1426.

Kiyonori Miura; Department of Obstetrics and Gynecology, Nagasaki University Graduate School of Biomedical Sciences, 1-7-1 Sakamoto, Nagasaki 852-8501, Japan; Email: kiyonori@nagasaki-u.ac.jp; Phone: +81-95-819-7363; Fax: +81-95-819-7365.

**Fig.S1 Duration from GT to RRSO of all cases.**

**Text summary**

The median time from GT to RRSO was 0.66 years (15 days to 18.5 years) from March 2006 to August 2021 for all criteria. (.docx)

**Table S1 Timing of RRSO and RRM for breast cancer patients**

**Text summary**

Of the 127 patients who underwent both RRSO and RRM, 69 (54.3%) performed both procedures at 1-month intervals, 31 (24.4%) performed RRSO followed by RRM, and 27 (21.3%) followed RRSO and RRM.

**
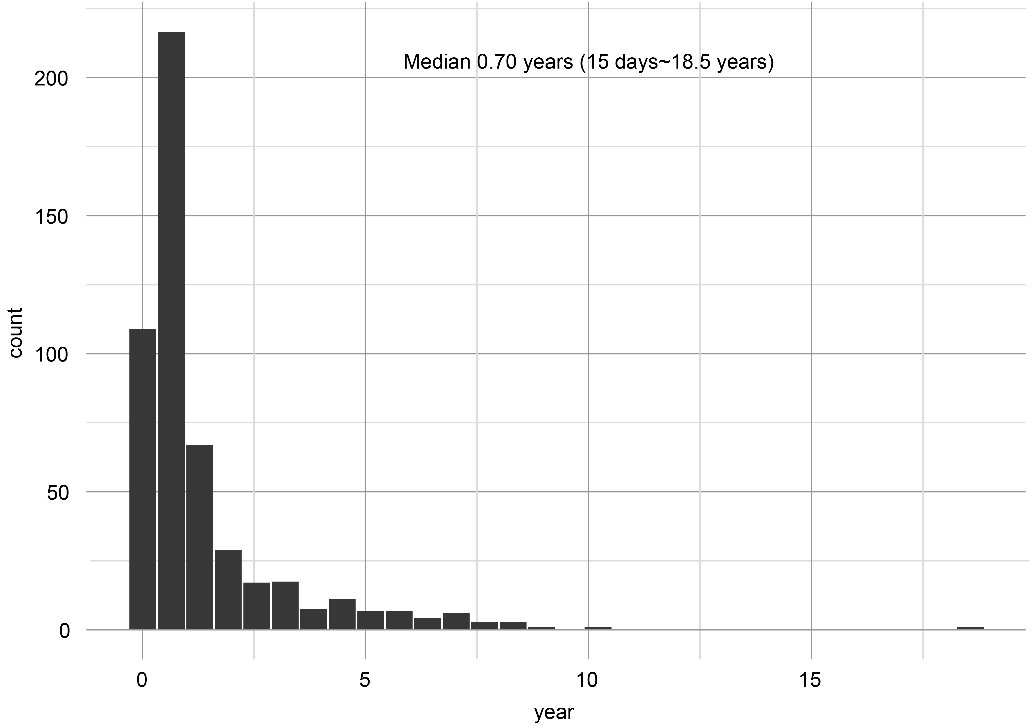
**

**Fig. S1** **Duration from genetic test to RRSO of all clients**

**Table S1 Timing of RRSO and RRM for breast cancer patients**

*1 Pre+Post : RRSO and RRM were performed separately during the pre and post periods

*2 RRSO+RRM : RRSO and RRM were performed within 1 month
